# Accelerated Orthodontics: A Descriptive Bibliometric Analysis of the Top 50 Cited Articles from 2012 to 2023

**DOI:** 10.3390/clinpract14050137

**Published:** 2024-08-29

**Authors:** Ahmed A. Alsulaiman, Osama A. Alsulaiman

**Affiliations:** Department of Preventive Dental Sciences, College of Dentistry, Imam Abdulrahman Bin Faisal University, Dammam 32222, Saudi Arabia; oaalsulaiman@iau.edu.sa

**Keywords:** accelerated orthodontics, bibliometric analysis, citation analysis, dental research, Scopus, COVID-19

## Abstract

Background and Objectives: Accelerated orthodontics represents a significant shift in dental practice aimed at reducing treatment times while maintaining optimal patient outcomes. This bibliometric analysis evaluated the research landscape of accelerated orthodontics from 2012 to 2023, focusing on publication trends, citation patterns, influential journals, leading institutions, and key contributors. Materials and Methods: A comprehensive search in Scopus identified 600 relevant articles, with the top 50 most-cited papers encompassing systematic reviews, randomized controlled trials, and experimental studies. Key techniques, such as corticotomy and piezocision, have been frequently highlighted for their effectiveness in expediting tooth movement. Results: The analysis revealed fluctuating annual scientific outputs, with notable peaks driven by technological advancements and increased patient demand for quicker orthodontic solutions. However, the production of high-impact papers was hindered by delays in citation accumulation and disruptions caused by the COVID-19 pandemic. Keyword analysis identified critical themes, such as orthodontic tooth movement, malocclusion, and demographic factors, while a global collaboration map underscored extensive international research partnerships. Leading journals included the American Journal of Orthodontics and Dentofacial Orthopedics, and prominent institutions such as the University of California at Los Angeles played significant roles in advancing the field. Conclusions: This study provides a comprehensive overview of the current state of accelerated orthodontics, emphasizing the need for continued research, particularly RCTs, to further refine and validate accelerated orthodontic techniques and improve clinical outcomes.

## 1. Introduction

Accelerated orthodontics represents a paradigm shift in dental practice aimed at reducing treatment times while maintaining optimal patient outcomes [1,2]. This field, which is rapidly advancing, has garnered extensive interest from researchers, healthcare professionals, and industry leaders who are looking for new ways to improve orthodontic treatment. Orthodontic treatment traditionally spans several months to years, necessitating regular clinical visits and patient compliance with treatment protocols [2,3,4].

Adult patients often hesitate to undergo orthodontic treatment because of concerns about the lengthy treatment duration, discomfort, and inconvenience associated with wearing orthodontic appliances [5]. A major problem for adults undergoing orthodontic treatment is the prolonged duration of the process. Adults typically prefer shorter treatment times and often require more esthetically pleasing appliances. Recent studies have revealed an increased number of adults in the United Kingdom seeking orthodontic care [6]. A notable study revealed that the primary motivation behind adults seeking orthodontic treatment is to achieve optimal dental alignment and enhance the esthetics of their smiles [7]. It is worth mentioning that orthodontic treatment for adults is significantly different from that for adolescents and is influenced by both psychological factors and biological considerations [8]. However, accelerated orthodontics introduces techniques aimed at expediting tooth movement and reducing the overall treatment duration. These techniques include surgical interventions such as corticotomy, piezocision, and the use of biological agents such as bone morphogenetic proteins (BMPs) [9,10,11,12]. The potential benefits of accelerated orthodontics include shorter treatment times, reduced patient discomfort, and improved treatment predictability, making it an appealing option for both patients and practitioners.

Accelerated orthodontics is a treatment option that promises quicker results; however, it presents some significant drawbacks. A key concern is the increased risk of root resorption, which can compromise the long-term health and stability of the teeth [13,14]. Additionally, the use of advanced methods such as corticotomies or bone remodeling devices often results in increased discomfort, pain, and potential complications. These techniques also come with a higher price tag, limiting accessibility [15,16,17]. Furthermore, the long-term effectiveness and safety of accelerated orthodontics is not as well-established as traditional methods, casting doubt on the durability of rapid treatments. Finally, the specialized training and equipment required to perform this treatment restrict its availability to certain practitioners, limiting patient options [16,18].

This study was conducted to accomplish several objectives through a bibliometric analysis of the top 50 papers on accelerated orthodontics. The primary aim was to identify the key contributors and institutions driving research in this particular field. Another objective was to evaluate publication trends over time and across diverse geographic regions. In addition, the analysis aimed to identify the most influential studies and journals in the field. Finally, the study sought to uncover emerging themes and gaps in the current research. By systematically reviewing the literature, this study aimed to provide a comprehensive understanding of the current state of accelerated orthodontic research. The ultimate goal of this analysis was to inform clinicians, researchers, and policymakers about the current state of accelerated orthodontics and guide future research priorities and clinical advancements in the field.

## 2. Materials and Methods

### 2.1. Data Sources and Search Strategies

Bibliometric analysis was conducted using Scopus as the sole data source. A comprehensive search strategy was used to identify relevant articles published between 2012 and 2023. The search was conducted on 30 June 2024 and was carefully designed to encompass all relevant studies on accelerated orthodontics, ensuring a thorough and exhaustive collection of data. The search results were exported as a BibTeX file for further analysis. The resulting dataset was analyzed to extract key bibliometric indicators, including the number of publications, citation counts, and the distribution of study types among the top 50 most-cited articles. The detailed search strategy is included in Appendix A. 

### 2.2. Inclusion Criteria

The analysis included all peer-reviewed publications related to accelerated orthodontics published between 2012 and 2023. This encompasses various study types, including original research articles, review articles, experimental studies, randomized controlled trials (RCTs), systematic reviews, cross-sectional studies, and retrospective studies. Only articles available in English and indexed in Scopus were considered to ensure the consistency and reliability of the data. Duplicate records, conference abstracts, and non-peer-reviewed articles were excluded to maintain the dataset quality. This inclusive approach ensured a comprehensive overview of the significant research contributions and trends in accelerated orthodontics over a specified period.

### 2.3. Data Collection

To ensure the accuracy and reliability of the data collection process, two independent reviewers meticulously verified the data entry, and considered a number of different data elements, including titles, keywords, publication dates, authors, linked organizations, publishing journals, the total number of citations, and the authors’ national and regional locations. This rigorous verification process helped to eliminate errors and inconsistencies, ensuring the integrity of the dataset used for the bibliometric analysis.

### 2.4. Statistical Analysis

The analysis was conducted using R version 4.3.3 and the bibliometrix package. Additionally, “biblioshiny”, a web interface for bibliometrix, was integrated into RStudio version 3.6.0, facilitating a multifaceted bibliometric analysis [19]. This comprehensive analysis included several key aspects: annual scientific production, average citations per year, most relevant sources, most relevant affiliations, keyword analysis, most relevant authors, author production over time, and a countries’ collaboration world map. The use of the bibliometrix package and biblioshiny interface enabled detailed and robust statistical analysis, providing valuable insights into the trends and patterns within the field of accelerated orthodontics from 2012 to 2023.

## 3. Results

**Figure 1:**
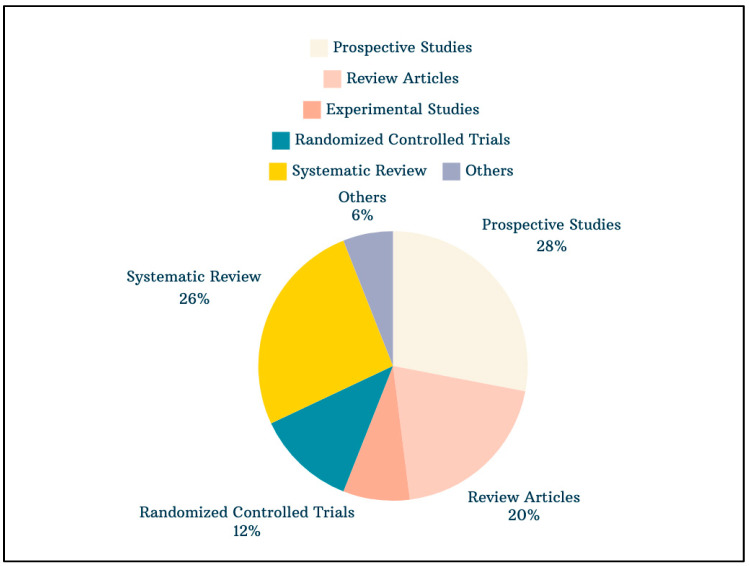
Distribution of study types among the top 50 most-cited articles in accelerated orthodontics (2012–2023).

A comprehensive search of studies on accelerated orthodontics yielded 846 papers. When the search was limited to publications published between 2012 and 2023, the number of studies was reduced to 600. Among the top 50 most-cited articles, 251 authors contributed to the publications with an average of 59.62 citations per document. The distribution of study types among the top 50 articles included 13 systematic reviews, 14 prospective studies, 6 randomized controlled trials, 4 experimental studies, 10 reviews, 1 case report, 1 cross-sectional study, and 1 preliminary study (Figure 1). Table 1 provides a detailed list of the top 50 cited papers, organized from the most-cited to the least-cited, with the total number of citations amounting to 2981. A table listing the top 50 most-cited original articles, excluding review articles, is included in Table S1.

### 3.1. Annual Scientific Production

**Figure 2:**
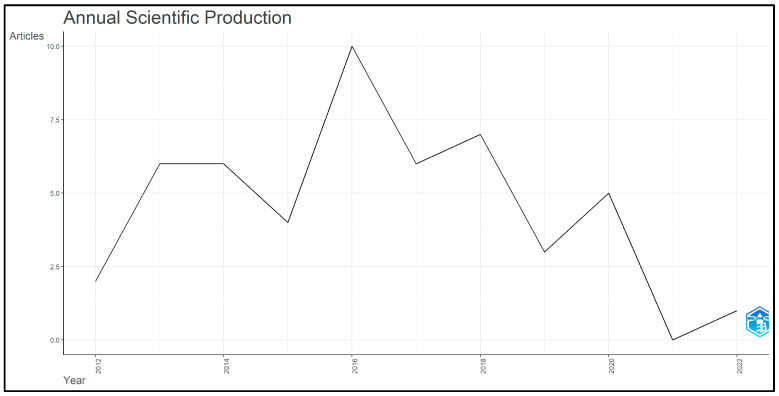
Annual scientific production of top 50 most-cited articles on accelerated orthodontics (2012–2023).

The annual scientific production of articles on accelerated orthodontics among the top 50 most-cited papers from 2012 to 2023 has exhibited a fluctuating trend. Starting with two articles in 2012, the number increased to six in both 2013 and 2014. There was a slight decline to four articles in 2015, followed by a peak of ten articles in 2016. The production then fluctuated, with six articles in 2017 and seven in 2018. A notable decline occurred in 2019 with only three articles, followed by a slight increase to five articles in 2020. However, there was a significant drop to zero articles in 2021 and only one article in 2022. This trend is illustrated in Figure 2, which shows the yearly distribution of articles and their corresponding scientific production.

### 3.2. Average Citation Per Year

**Figure 3:**
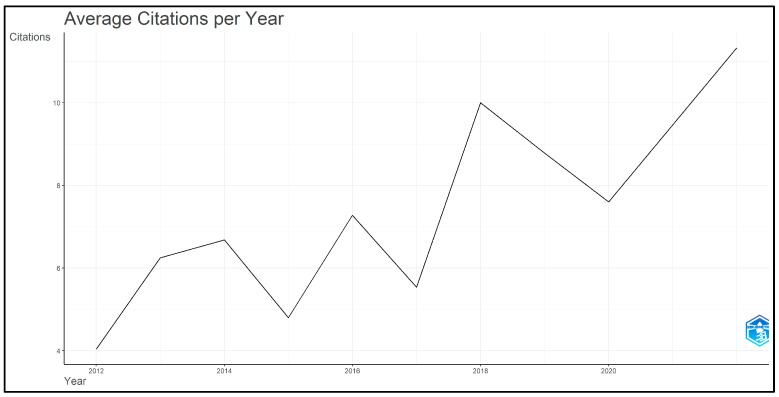
Average citations per year for top 50 most-cited articles on accelerated orthodontics (2012–2023).

The average number of citations per year for the top 50 most-cited articles on accelerated orthodontics from 2012 to 2023 demonstrated varying trends in scholarly impact (Figure 3). In 2012, the mean total citations per article (Mean TC per Art) was 52.5, with a mean citation per year (Mean TC per Year) of 4.04 over 13 citable years. In 2013, these values increased to 75 and 6.25, respectively, over the 12 citable years. The trend continued in 2014, with a Mean TC per Art of 73.5 and a Mean TC per year of 6.68, over 11 citable years. A decline was observed in 2015 with Mean TC per art at 48 and Mean TC per year at 4.80, over 10 citable years. However, there was a peak in 2016, with Mean TC per Art at 65.5, and Mean TC per Year at 7.28, over nine citable years. The trend dipped in 2017, with Mean TC per Art at 44.33, and Mean TC per Year at 5.54, over eight citable years. Then, 2018 saw an increase, with Mean TC per Art at 70 and Mean TC per Year at 10.00 over seven citable years, followed by 2019, with Mean TC per Art at 52.67 and Mean TC per Year at 8.78 over six citable years. In 2020, the values were 38 and 7.60, respectively, over five citable years. The year 2022 had the lowest Mean TC per art at 34, but a high Mean TC per year at 11.33, over three citable years.

**Figure 4:**
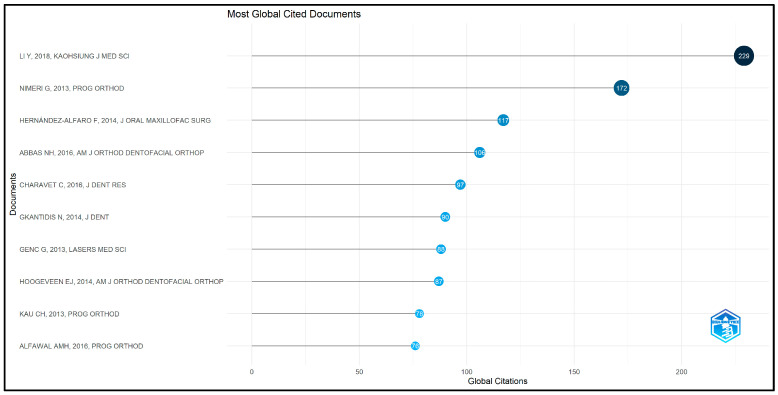
Top ten globally cited documents in accelerated orthodontics [15,16,20,21,22,23,24,25,26,27].

Among the top 50 most-cited articles, the top 10 most-cited documents were particularly notable for their significant contributions to the field. Leading this group is the article by Li (2018) [20] with 229 citations and an average of 32.71 citations per year, reflecting its substantial impact. Following closely are Nimeri G (2013) [15] with 172 citations and Hernández-Alfaro F (2014) [21] with 117 citations, showcasing their critical roles in advancing research on accelerated orthodontics. The top 10 most-cited documents globally among the top 50 cited articles are shown in Figure 4.

### 3.3. Most Relevant Sources

**Figure 5:**
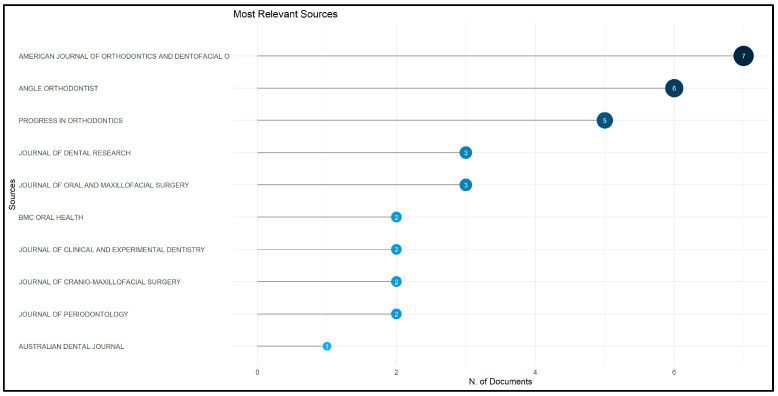
Distribution of most relevant sources in accelerated orthodontics literature (2012–2023).

The most relevant sources for the articles on accelerated orthodontics from 2012 to 2023 were identified, highlighting the key journals contributing significantly to this field. Leading the list is the American Journal of Orthodontics and Dentofacial Orthopedics, with 7 articles among the top 50 most-cited. This is followed by the Angle Orthodontist, contributing six articles, and Progress in Orthodontics, contributing five articles. Other notable journals include the Journal of Dental Research and the Journal of Oral and Maxillofacial Surgery, each contributing three articles. Additional relevant sources with two articles each are BMC Oral Health, Journal of Clinical and Experimental Dentistry, Journal of Cranio-Maxillofacial Surgery, and Journal of Periodontology. The distribution of these top 10 sources is illustrated in Figure 5, highlighting their prominence and contribution to the literature.

### 3.4. Most Relevant Affiliations

**Figure 6:**
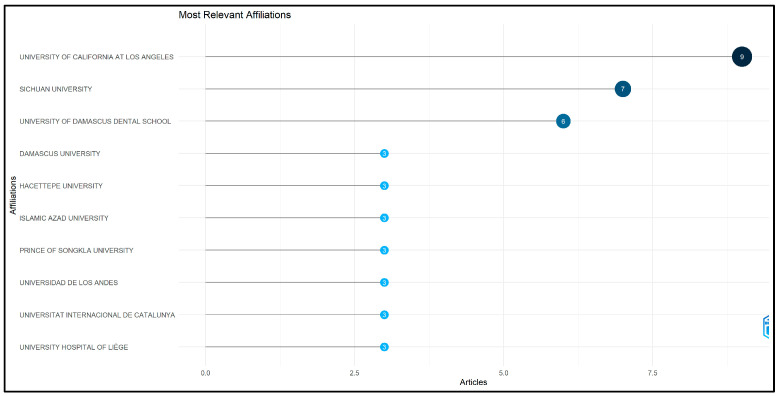
Most relevant affiliations among top 50 most-cited articles contributing to accelerated orthodontics research (2012–2023).

The most relevant affiliations contributing to research on accelerated orthodontics from 2012 to 2023 were identified, showcasing institutions that have made significant contributions to this specialized area. Topping the list is the University of California in Los Angeles, with 9 articles among the top 50 most-cited. Following closely are Sichuan University and University of Damascus Dental School, each contributing seven articles. Several institutions, including Damascus University, Hacettepe University, Islamic Azad University, Prince of Songkla University, Universidad de los Andes, Universitat Internacional de Catalunya, and University Hospital of Liège, have contributed three articles each. These affiliations, highlighted in Figure 6, underscore their substantial role in advancing research and innovation in accelerated orthodontics and in shaping the landscape of scholarly discourse in the field.

### 3.5. Keyword Analysis

**Figure 7:**
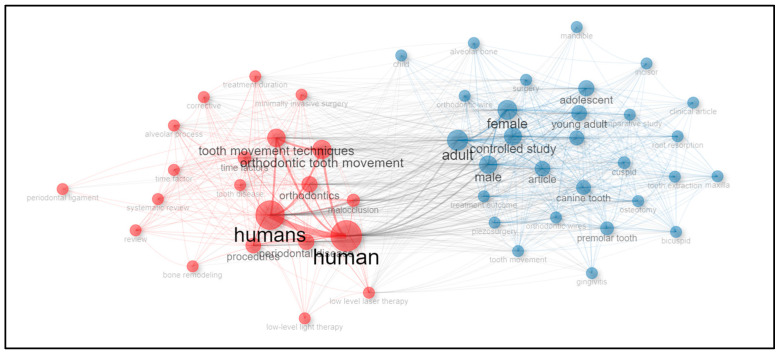
Co-occurrence network of keywords in accelerated orthodontics research.

**Figure 8:**
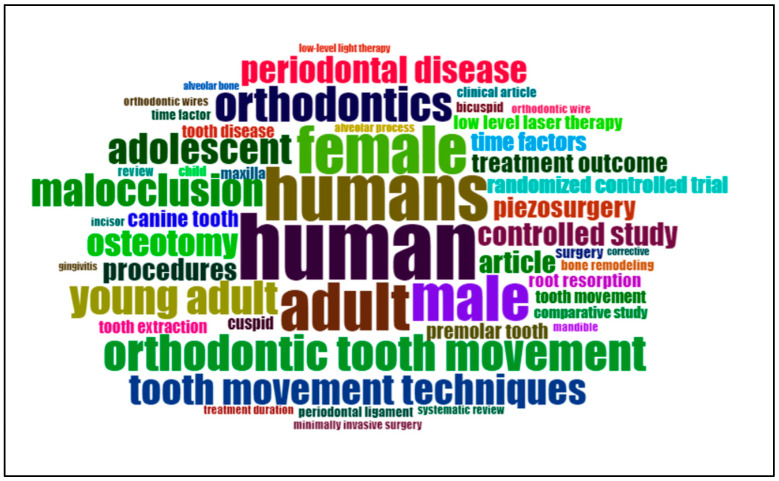
Word cloud representation of keyword frequencies in accelerated orthodontics Top 50 most-cited papers.

An analysis of keywords from the top 50 most-cited articles in accelerated orthodontics research between 2012 and 2023 revealed significant themes and focal points. Terms such as “human” (51 occurrences), “humans” (41 occurrences), “adult” (37 occurrences), “female” (36 occurrences), and “male” (35 occurrences) underscored the demographic focus and gender considerations of the studies. Key topics included “orthodontic tooth movement” (28 occurrences), “orthodontics” (27 occurrences), “young adult” (26 occurrences), “malocclusion” (25 occurrences), and “adolescent” (24 occurrences), highlighting the central areas of investigation and treatment approaches. Additionally, terms such as “osteotomy” (21 occurrences), “periodontal disease” (21 occurrences), “controlled study” (18 occurrences), and “procedures” (17 occurrences) reflect methodological and clinical aspects explored in the literature. These findings were further elucidated through a co-occurrence network (Figure 7) and visual representation in a word cloud (Figure 8), providing insights into the interconnected themes and research emphasis in accelerated orthodontics.

### 3.6. Most Relevant Authors and Author Production over Time

**Figure 9:**
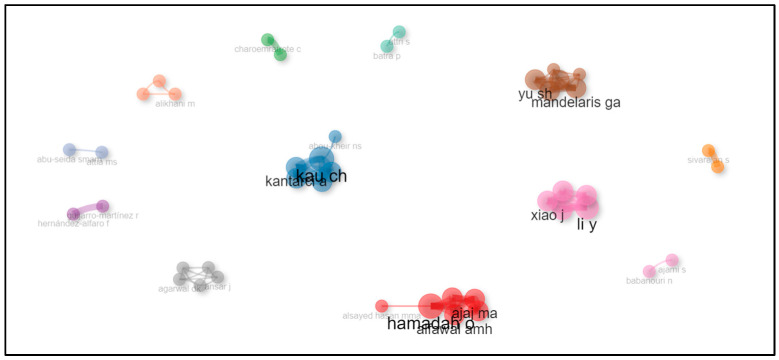
Collaborative networks of authors in accelerated orthodontics research.

The analysis of the top authors in accelerated orthodontics research from 2012 to 2023 highlights notable contributors and their production trends. Among the top 50 cited articles, Hamadah O, Kau CH, and Li Y stood out with significant contributions, each having authored multiple impactful papers. Hamadah O and Kau CH each contributed three articles, showcasing their consistent presence and impact in the field. Li Y, known for their influential work, particularly in 2018 with a highly cited article, also demonstrates a strong publication record. Author production over time reveals dynamic trends, with some authors such as Ajjaj MA, Alfawal AMH, and Charoemratrote C publishing consistently across different years, reflecting sustained research output and influence. These insights underscore the pivotal role of these authors in advancing knowledge and innovation in accelerated orthodontics. Additionally, their collaborative networks and interactions within the field are shown in Figure 9, which provides a comprehensive overview of their scholarly connections and collaborative efforts in accelerated orthodontic research. These analyses underscore the dynamic contributions of individual authors and their collaborative networks in advancing knowledge and innovation in the field.

### 3.7. Countries’ Collaboration World Map

**Figure 10:**
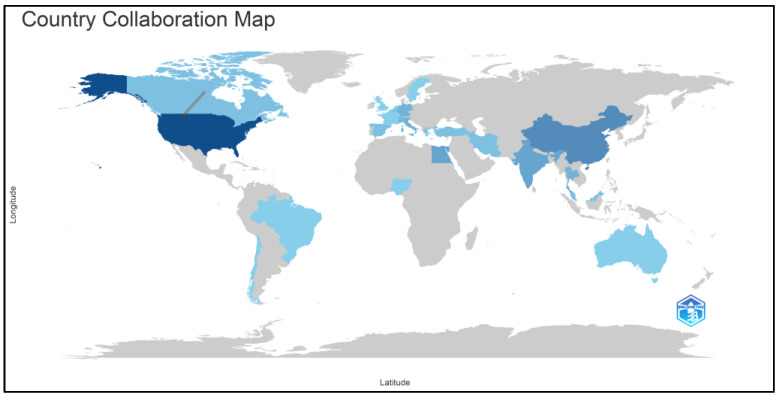
Countries’ collaboration network in accelerated orthodontics research (2012–2023).

The collaboration network among countries in accelerated orthodontic research from 2012 to 2023 reveals a diverse and interconnected landscape. Collaborations such as US–Canada, US–Belgium, and US–Thailand demonstrate transcontinental partnerships that contribute to global research endeavors. Similarly, collaborations between Malaysia and Switzerland and Malaysia–United Kingdom highlight international cooperation bridging different continents. These collaborative efforts underscore the global nature of research in accelerated orthodontics, facilitating knowledge exchange and mutual advancement across borders. The geographical distribution and interrelations of these collaborations are visually represented in Figure 10, illustrating the interconnectedness and impact of international partnerships in shaping the field’s scholarly discourse.

## 4. Discussion

The field of accelerated orthodontics has gained considerable attention over the past decade owing to its potential to reduce treatment times and improve patient satisfaction. This bibliometric analysis aimed to systematically evaluate the research landscape of accelerated orthodontics from 2012 to 2023. It focuses on publication trends, citation patterns, influential journals, leading institutions, and key contributors. By quantifying the annual scientific output and analyzing the average citations per year, we identified the most-cited papers and categorized them by study type. The analysis highlights the top 50 papers on this topic, underscores the significant role of key journals, such as the American Journal of Orthodontics and Dentofacial Orthopedics, and identifies leading institutions, such as the University of California at Los Angeles. Additionally, keyword analysis revealed dominant themes and focus areas, while author contributions and collaboration networks illustrated the extent of research partnerships. 

A thorough search for studies on accelerated orthodontics resulted in 846 papers, which narrowed down to 600 when focusing on publications from 2012 to 2023. Among the top 50 most-cited articles, 251 authors contributed, with an average of nearly 60 citations per document. These top articles included a mix of systematic reviews, prospective studies, randomized controlled trials, experimental studies, reviews, case reports, cross-sectional studies, and preliminary studies. The annual scientific production of these articles showed a fluctuating trend, peaking and declining over time. The average citations per year for these top articles varied, reflecting different levels of scholarly impact. The most relevant sources in this field were journals such as the American Journal of Orthodontics and Dentofacial Orthopedics, followed by the Angle Orthodontist and Progress in Orthodontics. The leading institutions included the University of California at Los Angeles and Sichuan University. Keyword analysis highlighted significant themes and focal points, such as demographics, orthodontic tooth movement, and clinical procedures. Notable authors in the field included Hamadah O, Kau CH, and Li Y, each contributing multiple influential papers. The research landscape also featured extensive international collaboration, with notable partnerships between countries such as the USA and Canada, Malaysia and Switzerland, and others, underscoring the global nature of research in accelerated orthodontics.

Among the top 50 articles on accelerated orthodontics, most focused on the mechanisms underlying orthodontic procedures. Some were randomized controlled trials, whereas others were systematic reviews and meta-analyses. This diversity highlights the importance of future research, particularly emphasizing randomized controlled trials, to further advance this field. Various techniques have been employed to shorten the duration of orthodontic treatment. However, surgical interventions tend to be more clinically effective, often yielding better results in reducing the overall treatment duration [27]. Kole’s surgical procedure proposes that accelerated tooth movement is achieved by selectively cutting the bone, allowing the movement of “blocks of bone” [68]. However, recent evidence indicates that the increased rate of tooth movement is due to localized osteoporosis as part of the healing process, known as the regional acceleratory phenomenon (RAP) [69]. Various surgical techniques have been employed to leverage the RAP and accelerate tooth movement, including conventional corticotomy [70,71], piezocision-based flapless corticotomy [39,72], corticision [73], and laser-assisted flapless corticotomy [74,75].

The general pattern of the annual scientific output demonstrates a downward trend in publications on accelerated orthodontics, with a notable peak of 80 articles in 2016. This increase suggests that there is a slight interest and investment in the field, which is probably driven by technological advancements and the growing demand for quicker orthodontic solutions. However, the decline in publications after 2019 might indicate a change in research priorities, market saturation, or the influence of global events, such as the COVID-19 pandemic, on research and clinical activities [76,77]. The production of high-impact papers in accelerated orthodontics is limited by several factors. First, scientific papers typically require a considerable amount of time to receive citations, especially if they are not published in prestigious, high-impact journals that naturally attract more attention and citations. This time lag means that even high-quality research may not be immediately recognized or widely cited. Second, the COVID-19 pandemic has had a profound impact on global research activities, including orthodontics. During the pandemic, many research projects in orthodontics were halted or delayed, owing to restrictions on in-person activities and access to laboratories and clinical settings. Additionally, the focus of many researchers and healthcare professionals has shifted towards addressing the immediate challenges posed by the pandemic, further slowing the progress of ongoing research in fields such as orthodontics. Moreover, the pandemic has disrupted the usual channels of academic communication and collaboration. Conferences and other professional gatherings where researchers typically share and discuss their findings were canceled or moved online, reducing opportunities for networking and the dissemination of new research. This disruption likely contributed to the slower pace of publication and reduced the visibility of new research during this period. A recent investigation revealed that the COVID-19 pandemic had a considerable influence on orthodontic clinical education in both pre- and post-doctoral orthodontic programs [78]. In general, there was a noticeable decrease in patient care, which could result in the delayed treatment of patients and fewer training opportunities for learners.

The keyword analysis results revealed important themes, including “orthodontic tooth movement”, “malocclusion”, and “maxilla”, which are central to the research focus in this field. Additionally, the presence of demographic terms such as “human”, “male”, and “female” highlights the diverse demographics involved in studies, which is crucial for generalizing findings across different populations. The co-occurrence network provides insights into how these themes intersect, emphasizing the need for comprehensive research frameworks and multidisciplinary approaches to address various aspects of accelerated orthodontics. The international collaboration map highlights a strong global network, with notable partnerships between countries such as the USA, Brazil, Italy, and others. These collaborations facilitate knowledge exchange, resource sharing, and diverse perspectives, thereby enhancing the quality and impact of research. The extent of the network indicates that accelerated orthodontics is a globally relevant field, with significant contributions from various regions fostering innovation and advancements.

A widely implemented intervention in accelerated orthodontics is the use of PAOO, which has been the subject of extensive research [4]. Studies suggest that both PAOO and corticotomy-only groups experience a reduction in bone density [79,80]. This decrease is linked to the surgical trauma that triggers localized osteoporosis. However, further research has shown a substantial increase in alveolar bone density during the follow-up phase. PAOO has been shown to significantly reduce treatment time compared to alternative methods [81].

Studies have established the usefulness of PAOO in hastening the process of tooth alignment and leveling [82,83]. Additionally, studies have emphasized their effectiveness in accelerating the retraction of the upper anterior teeth compared to conventional orthodontic methods [49,84]. This expedited retraction is attributed to the surgical procedure, which triggers the regional accelerator phenomenon (RAP). This phenomenon takes effect shortly after surgery and reaches its peak within one–two months. RAP can persist for half to two years, with its maximum observed approximately one month after surgery, followed by a gradual decline over subsequent months [85]. 

This bibliography represents the first significant contribution to the literature on accelerated orthodontics and offers fundamental insights into this field. It incorporates a diverse range of recent and relevant sources, providing a well-structured organization that enables seamless access to specific studies or themes. The inclusion of various viewpoints and methodologies enriches comprehension, presenting a sophisticated outlook on the subject matter. Appropriate citation ensures transparency, allowing for the further verification and exploration of the referenced research. Furthermore, this bibliometric analysis explains the top 50 articles and lays the foundation for future studies on high-impact work. However, the bibliography’s reliance exclusively on the Scopus database and its restriction to the timeframe between 2012 and 2023 may introduce bias. It could potentially disregard relevant studies not indexed in Scopus or published beyond this period, limiting the completeness of coverage. Although comprehensive within its scope, the literature may not encompass all aspects of accelerated orthodontic research, particularly those from alternative databases or older studies. These factors could potentially hamper the breadth and depth of the insights offered, impacting the overall perspective on the topic.

## 5. Conclusions

In conclusion, accelerated orthodontics represents a transformative approach in dental practice, focusing on reducing treatment duration while ensuring optimal outcomes. Our bibliometric analysis, covering research from 2012 to 2023, identified key trends, influential studies, and major contributors to the field. It reveals a diverse range of studies, including systematic reviews, randomized controlled trials, and experimental research, highlighting the significant role of surgical techniques such as corticotomy and piezocision in expediting tooth movement. Despite the fluctuating trend in annual scientific output, with peaks in interest driven by technological advancements and patient demand for quicker solutions, the production of high-impact papers has faced challenges, particularly owing to the time required for citation accumulation and disruptions caused by the COVID-19 pandemic. The keyword analysis underscores critical themes such as orthodontic tooth movement and demographic considerations, while international collaborations reflect a robust global research network. The findings of this study provide a comprehensive overview of accelerated orthodontics, emphasizing the importance of ongoing research, especially randomized controlled trials, to further advance this field.

## Figures and Tables

**Table 1:** List of top 50 most-cited articles in accelerated orthodontics (2012–2023), ranked by total citations.

Title	First Author, Year	Journal Name	Total Citations	TC per Year	Normalized TC	Study Type
Orthodontic tooth movement: The biology and clinical implications [20]	LI Y, 2018	The Kaohsiung journal of medical sciences	229	32.71	3.27	Review
Acceleration of tooth movement during orthodontic treatment—a frontier in Orthodontics [15]	NIMERI G, 2013	Progress in Orthodontics	172	14.33	2.29	Review
Surgery First in Orthognathic Surgery: What Have We Learned? A Comprehensive Workflow Based on 45 Consecutive Cases [21]	HERNÁNDEZ-ALFARO F, 2014	Journal of Oral and Maxillofacial Surgery	117	10.64	1.59	Prospective Study
Evaluation of corticotomy-facilitated orthodontics and piezocision in rapid canine retraction [22]	ABBAS NH, 2016	American Journal of Orthodontics and Dentofacial Orthopedics	106	11.78	1.62	Prospective Study
Localized Piezoelectric Alveolar Decortication for Orthodontic Treatment in Adults: A Randomized Controlled Trial [16]	CHARAVET C, 2016	Journal of Dental Research	97	10.78	1.48	Randomized Controlled Trial
Effectiveness of non-conventional methods for accelerated orthodontic tooth movement: A systematic review and meta-analysis [23]	GKANTIDIS N, 2014	Journal of Dentistry	90	8.18	1.22	Systematic Review
Effect of low-level laser therapy (LLLT) on orthodontic tooth movement [24]	GENC G, 2013	Lasers in Medical Science	88	7.33	1.17	Prospective Study
Surgically facilitated orthodontic treatment: A systematic review [25]	HOOGEVEEN EJ, 2014	American Journal of Orthodontics and Dentofacial Orthopedics	87	7.91	1.18	Systematic Review
Photobiomodulation accelerates orthodontic alignment in the early phase of treatment [26]	KAU CH, 2013	Progress in Orthodontics	78	6.50	1.04	Prospective Study
Effectiveness of minimally invasive surgical procedures in the acceleration of tooth movement: a systematic review and meta-analysis [27]	ALFAWAL AMH, 2016	Progress in Orthodontics	76	8.44	1.16	Systematic Review
Corticotomies and Orthodontic Tooth Movement: A Systematic Review [28]	PATTERSON BM, 2016	Journal of Oral and Maxillofacial Surgery	75	8.33	1.15	Systematic Review
Accelerated tooth movement with piezocision and its periodontal-transversal effects in patients with Class II malocclusion [29]	AKSAKALLI S, 2016	The Angle orthodontist	71	7.89	1.08	Prospective Study
Force-induced Adrb2 in Periodontal Ligament Cells Promotes Tooth Movement [30]	CAO H, 2014	Journal of Dental Research	68	6.18	0.93	Experimental study
Periodontally accelerated osteogenic orthodontics (PAOO)—a review [31]	AMIT G, 2012	Journal of clinical and experimental dentistry	64	4.92	1.22	Review
Low-level laser therapy effectiveness in accelerating orthodontic tooth movement: A randomized controlled clinical trial [32]	ALSAYED HASAN MMA, 2017	The Angle orthodontist	63	7.88	1.42	Randomized Controlled Trial
Efficacy of surgical and non-surgical interventions on accelerating orthodontic tooth movement: a systematic review [33]	KALEMAJ Z, 2015	European journal of oral implantology	61	6.10	1.27	Systematic Review
Mini-implant supported canine retraction with micro-osteoperforation: A split-mouth randomized clinical trial [34]	SIVARAJAN S, 2019	The Angle orthodontist	60	10.00	1.14	Randomized Controlled Trial
Vibratory stimulation increases interleukin-1 beta secretion during orthodontic tooth movement [35]	LEETHANAKUL C, 2016	The Angle orthodontist	57	6.33	0.87	Prospective Study
Growth modification of the face: A current perspective with emphasis on Class III treatment [36]	DE CLERCK HJ, 2015	American Journal of Orthodontics and Dentofacial Orthopedics	56	5.60	1.17	Review
Efficacy of piezocision on accelerating orthodontic tooth movement: A systematic review [37]	YI J, 2017	The Angle orthodontist	56	7.00	1.26	Systematic Review
Effect of micro-osteoperforation on the rate of canine retraction: a split-mouth randomized controlled trial [38]	ABOALNAGA AA, 2019	Progress in Orthodontics	55	9.17	1.04	Randomized Controlled Trial
Evaluation of piezocision and laser-assisted flapless corticotomy in the acceleration of canine retraction: a randomized controlled trial [39]	ALFAWAL AMH, 2018	Head & face medicine	54	7.71	0.77	Systematic Review
American Academy of Periodontology best evidence consensus statement on modifying periodontal phenotype in preparation for orthodontic and restorative treatment [40]	KAO RT, 2020	Journal of periodontology	54	10.80	1.42	Review
Ability of mini-implant-facilitated micro-osteoperforations to accelerate tooth movement in rats [41]	CHEUNG T, 2016	American journal of orthodontics and dentofacial orthopedics	53	5.89	0.81	Experimental study
Comparison of rate of tooth movement and pain perception during accelerated tooth movement associated with conventional fixed appliances with micro-osteoperforations—a **randomised** controlled trial [42]	ATTRI S, 2018	Journal of orthodontics	48	6.86	0.69	Randomized Controlled Trial
Efficacy of low-level laser therapy in accelerating tooth movement, preventing relapse and managing acute pain during orthodontic treatment in humans: a systematic review [43]	SONESSON M, 2016	BMC Oral Health volume	48	5.33	0.73	Systematic Review
Alveolar corticotomies for accelerated orthodontics: A systematic review [44]	GIL APS, 2018	Journal of Cranio-Maxillofacial Surgery	48	6.86	0.69	Systematic Review
Low-level laser therapy increases interleukin-1β in gingival crevicular fluid and enhances the rate of orthodontic tooth movement [45]	VARELLA AM, 2018	American Journal of Orthodontics and Dentofacial Orthopedics	45	6.43	0.64	Prospective Study
Micro-osteoperforations: Minimally invasive accelerated tooth movement [46]	ALIKHANI M, 2015	Seminars in Orthodontics	44	4.40	0.92	Review
Effectiveness of adjunctive interventions for accelerating orthodontic tooth movement: a systematic review of systematic reviewsb [47]	YI J, 2017	Journal of Oral Rehabilitation	44	5.50	0.99	Systematic Review
Effectiveness and Safety of Minimally Invasive Orthodontic Tooth Movement Acceleration: A Systematic Review and Meta-analysis [48]	FU T, 2019	Journal of Dental Research	43	7.17	0.82	Systematic Review
Assessment of Corticotomy Facilitated Tooth Movement and Changes in Alveolar Bone Thickness—A CT Scan Study [49]	BHATTACHARYA P, 2014	Journal of clinical and diagnostic research	42	3.82	0.57	Cross-sectional Study
Corticotomy-facilitated orthodontics in adults using a further modified technique [50]	SHOREIBAH EA, 2012	Journal of the International Academy of Periodontology	41	3.15	0.78	Prospective Study
Augmented Corticotomy Combined With Accelerated Orthodontic Forces in Class III Orthognathic Patients: Morphologic Aspects of the Mandibular Anterior Ridge With Cone-Beam Computed Tomography [51]	COSCIA G, 2013	Journal of Oral and Maxillofacial Surgery	40	3.33	0.53	Prospective Study
Intraoral photobiomodulation-induced orthodontic tooth alignment: a preliminary study [52]	SHAUGHNESSY T, 2016	BMC Oral Health	38	4.22	0.58	Preliminary Study
Is periodontal phenotype modification therapy beneficial for patients receiving orthodontic treatment? An American Academy of Periodontology best evidence review [53]	WANG CW, 2020	Journal of periodontology	38	7.60	1.00	Review
Comparing the 810 nm Diode Laser with Conventional Surgery in Orthodontic Soft Tissue Procedures [54]	IZE-IYAMU IN, 2013	Ghana medical journal	38	3.17	0.51	Prospective Study
Surgery-first approach in orthognathic surgery: Psychological and biological aspects—A prospective cohort study [55]	ZINGLER S, 2017	Journal of Cranio-Maxillofacial Surgery	38	4.75	0.86	Prospective Study
Interseptal bone reduction on the rate of maxillary canine retraction [56]	LEETHANAKUL C, 2014	The Angle orthodontist	37	3.36	0.50	Prospective Study
Accelerated orthodontic treatment—what’s the evidence? [57]	MILES P, 2017	Australian dental journal	35	4.38	0.79	Review
The use of micro-osteoperforation concept for accelerating differential tooth movement [58]	FEIZBAKHSH M, 2018	Journal of the World Federation of Orthodontists	34	4.86	0.49	Prospective Study
Sequential piezocision: A novel approach to accelerated orthodontic treatment [59]	KESER EI, 2013	American Journal of Orthodontics and Dentofacial Orthopedics	34	2.83	0.45	Case report
Is Orthodontic Treatment with Microperforations Worth It? A Scoping Review [60]	MASPERO C, 2022	Children (Basel, Switzerland)	34	11.33	1.00	Review
Use of leukocyte and platelet-rich fibrin (L-PRF) in periodontally accelerated osteogenic orthodontics (PAOO): Clinical effects on edema and pain [61]	MUÑOZ F, 2016	Journal of clinical and experimental dentistry	34	3.78	0.52	Prospective Study
Effects of Compressive and Tensile Strain on Macrophages during Simulated Orthodontic Tooth Movement [62]	SCHRÖDER A, 2020	Mediators of inflammation	34	6.80	0.89	Experimental study
The effect of micro-osteoperforations on the rate of orthodontic tooth movement: A systematic review and meta-analysis [63]	SIVARAJAN S, 2020	American Journal of Orthodontics and Dentofacial Orthopedics	34	6.80	0.89	Systematic Review
Periostin promotes migration, proliferation, and differentiation of human periodontal ligament mesenchymal stem cells [64]	WU Z, 2018	Connective tissue research	32	4.57	0.46	Experimental study
Osteoimmunology in orthodontic tooth movement [65]	JIANG C, 2015	Oral diseases	31	3.10	0.65	Review
Effect of mini-screw-facilitated micro-osteoperforation on the rate of orthodontic tooth movement: a single-center, split-mouth, randomized, controlled trial [66]	BABANOURI N, 2020	Progress in Orthodontics	30	6.00	0.79	Randomized Controlled Trial
Influence of piezotomy and osteoperforation of the alveolar process on the rate of orthodontic tooth movement: a systematic review [67]	HOFFMANN S, 2017	Journal of orofacial orthopedics	30	3.75	0.68	Systematic Review

## Data Availability

The original contributions presented in the study are included in the article and Supplementary Materials; further inquiries can be directed to the corresponding author/s.

## Supplementary Materials

The following supporting information can be downloaded at: https://www.mdpi.com/article/10.3390/clinpract14050137/s1, Table S1: List of Top 50 Most-Cited Articles in Accelerated Orthodontics (2012–2023), Ranked by Total Citations. (Original articles only, excluding reviews).

## Appendix A

Scopus search string:

(TITLE-ABS-KEY (accelerated AND orthodontics) OR TITLE-ABS-KEY (orthodontic AND acceleration) OR TITLE-ABS-KEY (accelerated AND tooth AND movement) OR TITLE-ABS-KEY (rapid AND orthodontic AND treatment) OR TITLE-ABS-KEY (speed AND orthodontics) OR TITLE-ABS-KEY (expedited AND orthodontics) OR TITLE-ABS-KEY (fast AND orthodontic AND treatment) OR TITLE-ABS-KEY (quick AND orthodontic AND treatment) OR TITLE-ABS-KEY (enhanced AND orthodontics) OR TITLE-ABS-KEY (accelerated AND dental AND movement) OR TITLE-ABS-KEY (rapid AND dental AND alignment) AND TITLE-ABS-KEY (micro-osteoperforation) OR TITLE-ABS-KEY (low-level AND laser AND therapy) OR TITLE-ABS-KEY (piezocision) OR TITLE-ABS-KEY (corticotomy) OR TITLE-ABS-KEY (vibration AND therapy) OR TITLE-ABS-KEY (high-frequency AND vibration) OR TITLE-ABS-KEY (photobiomodulation) OR TITLE-ABS-KEY (acceledent) OR TITLE-ABS-KEY (wilckodontics) OR TITLE-ABS-KEY (propel AND orthodontics) OR TITLE-ABS-KEY (pulsed AND electromagnetic AND field) OR TITLE-ABS-KEY (alveolar AND corticotomy) OR TITLE-ABS-KEY (surgical-assisted AND orthodontics) OR TITLE-ABS-KEY (laser-assisted AND orthodontics) OR TITLE-ABS-KEY (self-ligating AND brackets) OR TITLE-ABS-KEY (mops) OR TITLE-ABS-KEY (lllt) OR TITLE-ABS-KEY (paoo) OR TITLE-ABS-KEY (periodontally AND accelerated AND osteogenic AND orthodontics) OR TITLE-ABS-KEY (cytokine AND therapy) OR TITLE-ABS-KEY (drug-induced AND orthodontic AND acceleration) OR TITLE-ABS-KEY (mechanical AND vibration) OR TITLE-ABS-KEY (resonance AND vibration) OR TITLE-ABS-KEY (light AND emitting AND diodes) OR TITLE-ABS-KEY (magnetic AND fields) OR TITLE-ABS-KEY (corticostomy) OR TITLE-ABS-KEY (osteotomy) OR TITLE-ABS-KEY (piezoincision) OR TITLE-ABS-KEY (peridontally AND assisted) OR TITLE-ABS-KEY (osteogenic AND orthodontic AND treatment) OR TITLE-ABS-KEY (dental AND distraction) OR TITLE-ABS-KEY (dentoalveolar AND distraction AND osteogenesis) OR TITLE-ABS-KEY (calcium) OR TITLE-ABS-KEY (vitamin AND d3) OR TITLE-ABS-KEY (parathyroid) OR TITLE-ABS-KEY (prostaglandin) OR TITLE-ABS-KEY (corticosteroids) OR TITLE-ABS-KEY (osteocalcin) OR TITLE-ABS-KEY (cytokines) OR TITLE-ABS-KEY (relaxin)).
