# Supplementary material for: Accelerated Orthodontics: A Descriptive Bibliometric Analysis of the Top 50 Cited Articles from 2012 to 2023
Source: Clin Pract. 2024 Aug 29;14(5):1716–36. doi: 10.3390/clinpract14050137 (PMC11417789; doi:10.3390/clinpract14050137)
Supplement: Supplementary file 1 [file clinpract-14-00137-s001.zip › clinpract-3109786-supplementary.pdf]

Table S1

List of Top 50 Most-Cited Articles in Accelerated Orthodontics (2012-2023), Ranked by Total Citations. (original articles only, excluding reviews).

DOI	Title	First author, Year	Journal Name	Total Citations	TC per Year	Normalized TC	Study type
10.1016/j.joms.2013.08.013	Surgery First in Orthognathic Surgery: What Have We Learned? A Comprehensive Workflow Based on 45 Consecutive Cases	HERNÁNDEZ-ALFARO F, 2014	Journal of Oral and Maxillofacial Surgery	117	10.64	1.59	Prospective Study
10.1016/j.ajodo.2015.09.029	Evaluation of corticotomy-facilitated orthodontics and piezocision in rapid canine retraction	ABBAS NH, 2016	American Journal of Orthodontics and Dentofacial Orthopedics	106	11.78	1.62	Prospective Study
10.1177/0022034516645066	Localized Piezoelectric Alveolar Decortication for Orthodontic Treatment in Adults: A Randomized Controlled Trial	CHARAVET C, 2016	Journal of Dental Research	97	10.78	1.48	Randomized Controlled Trial
10.1007/s10103-012-1059-6	Effect of low-level laser therapy (LLLT) on orthodontic tooth movement	GENC G, 2013	Lasers in Medical Science	88	7.33	1.17	Prospective Study
10.1186/2196-1042-14-30	Photobiomodulation accelerates orthodontic alignment in the early phase of treatment	KAU CH, 2013	Progress in Orthodontics	78	6.50	1.04	Prospective Study
10.2319/012215-49.1	Accelerated tooth movement with piezocision and its periodontal-transversal effects in patients with Class II malocclusion	AKSAKALLI S, 2016	The Angle orthodontist	71	7.89	1.08	Prospective Study
10.1177/0022034514551769	Force-induced Adrb2 in Periodontal Ligament Cells Promotes Tooth Movement	CAO H, 2014	Journal of Dental Research	68	6.18	0.93	Experimental study
10.2319/062716-503.1	Low-level laser therapy effectiveness in accelerating orthodontic tooth movement: A randomized controlled clinical trial	ALSAYED HASAN MMA, 2017	The Angle orthodontist	63	7.88	1.42	Randomized Controlled Trial
10.2319/011518-47.1	Mini-implant supported canine retraction with micro-osteoperforation: A split-mouth randomized clinical trial	SIVARAJAN S, 2019	The Angle orthodontist	60	10.00	1.14	Randomized

							Controlled Trial
10.2319/111914-830.1	Vibratory stimulation increases interleukin-1 beta secretion during orthodontic tooth movement	LEETHANAKUL C, 2016	The Angle orthodontist	57	6.33	0.87	Prospective Study
10.1186/s40510-019-0274-0	Effect of micro-osteoperforation on the rate of canine retraction: a split-mouth randomized controlled trial	ABOALNAGA AA, 2019	Progress in Orthodontics	55	9.17	1.04	Randomized Controlled Trial
10.1016/j.ajodo.2016.04.030	Ability of mini-implant-facilitated micro-osteoperforations to accelerate tooth movement in rats	CHEUNG T, 2016	American journal of orthodontics and dentofacial orthopedics	53	5.89	0.81	Experimental study
10.1080/14653125.2018.1528746	Comparison of rate of tooth movement and pain perception during accelerated tooth movement associated with conventional fixed appliances with micro-osteoperforations - a randomised controlled trial	ATTRI S, 2018	Journal of orthodontics	48	6.86	0.69	Randomized Controlled Trial
10.1016/j.ajodo.2018.01.012	Low-level laser therapy increases interleukin-1β in gingival crevicular fluid and enhances the rate of orthodontic tooth movement	VARELLA AM, 2018	American Journal of Orthodontics and Dentofacial Orthopedics	45	6.43	0.64	Prospective Study
10.7860/JCDR/2014/9448.4954	Assessment of Corticotomy Facilitated Tooth Movement and Changes in Alveolar Bone Thickness - A CT Scan Study	BHATTACHARYA P, 2014	Journal of clinical and diagnostic research	42	3.82	0.57	Cross-sectional Study
https://pubmed.ncbi.nlm.nih.gov/23210198/	Corticotomy-facilitated orthodontics in adults using a further modified technique	SHOREIBAH EA, 2012	Journal of the International Academy of Periodontology	41	3.15	0.78	Prospective Study
10.1016/j.joms.2013.04.022	Augmented Corticotomy Combined With Accelerated Orthodontic Forces in Class III Orthognathic Patients: Morphologic Aspects of the Mandibular Anterior Ridge With Cone-Beam Computed Tomography	COSCIA G, 2013	Journal of Oral and Maxillofacial Surgery	40	3.33	0.53	Prospective Study
10.1186/s12903-015-0159-7	Intraoral photobiomodulation-induced orthodontic tooth alignment: a preliminary study	SHAUGHNESSY T, 2016	BMC Oral Health	38	4.22	0.58	Preliminary Study
https://www.ncbi.nlm.nih.gov/pmc/articles/PMC3875278/	Comparing the 810nm Diode Laser with Conventional Surgery in Orthodontic Soft Tissue Procedures	IZE-IYAMU IN, 2013	Ghana medical journal	38	3.17	0.51	Prospective Study

10.1016/j.jcms.2017.05.031	Surgery-first approach in orthognathic surgery: Psychological and biological aspects – A prospective cohort study	ZINGLER S, 2017	Journal of Cranio-Maxillofacial Surgery	38	4.75	0.86	Prospective Study
10.2319/100613-737.1	Interseptal bone reduction on the rate of maxillary canine retraction	LEETHANAKUL C, 2014	The Angle orthodontist	37	3.36	0.50	Prospective Study
10.1016/j.ejwf.2018.04.002	The use of micro-osteoperforation concept for accelerating differential tooth movement	FEIZBAKHSH M, 2018	Journal of the World Federation of Orthodontists	34	4.86	0.49	Prospective Study
10.1016/j.ajodo.2012.12.014	Sequential piezocision: A novel approach to accelerated orthodontic treatment	KESER EI, 2013	American Journal of Orthodontics and Dentofacial Orthopedics	34	2.83	0.45	Case report
10.4317/jced.52760	Use of leukocyte and platelet-rich fibrin (L-PRF) in periodontally accelerated osteogenic orthodontics (PAOO): Clinical effects on edema and pain	MUÑOZ F, 2016	Journal of clinical and experimental dentistry	34	3.78	0.52	Prospective Study
10.1155/2020/2814015	Effects of Compressive and Tensile Strain on Macrophages during Simulated Orthodontic Tooth Movement	SCHRÖDER A, 2020	Mediators of inflammation	34	6.80	0.89	Experimental study
10.1080/03008207.2017.1306060	Periostin promotes migration, proliferation, and differentiation of human periodontal ligament mesenchymal stem cells	WU Z, 2018	Connective tissue research	32	4.57	0.46	Experimental study
10.1186/s40510-020-00306-8	Effect of mini-screw-facilitated micro-osteoperforation on the rate of orthodontic tooth movement: a single-center, split-mouth, randomized, controlled trial	BABANOURI N, 2020	Progress in Orthodontics	30	6.00	0.79	Randomized Controlled Trial
10.1111/ocr.12014	Fiberotomy enhances orthodontic tooth movement and diminishes relapse in a rat model	YOUNG L, 2013, ORTHODONT CRANIOFAC RES	Orthodontics & craniofacial research	28	2.33	1.54	Experimental study
10.4041/kjod.2017.47.2.130	The combined use of computer-guided, minimally invasive, flapless corticotomy and clear aligners as a novel approach to moderate crowding: A case report	CASSETTA M, 2017, KOREAN J ORTHOD	Korean journal of orthodontics	27	3.38	1.55	Case report
10.1016/j.ajodo.2013.12.021	Timing of force application affects the rate of tooth movement into surgical alveolar defects with grafts in beagles	AHN HW, 2014, AM J ORTHOD DENTOFACIAL ORTHOP	American journal of orthodontics and dentofacial orthopedics	27	2.45	1.42	Experimental study
10.1186/s40510-018-0244-y	Comparison of piezocision and discision methods in orthodontic treatment	YAVUZ MC, 2018, PROG ORTHOD	Progress in orthodontics	26	3.71	1.45	Prospective Study

10.2319/061714-439.1	Mandibular irregularity index stability following alveolar corticotomy and grafting: a 10-year preliminary study	MAKKI L, 2015, ANGLE ORTHOD	The Angle orthodontist	26	2.60	1.50	Preliminary Study
10.1007/s10103-016-2094-5	Decrowding of lower anterior segment with and without photobiomodulation: a single center, randomized clinical trial	NAHAS AZ, 2017, LASERS MED SCI	Lasers in medical science	24	3.00	1.38	Randomized Controlled Trial
10.1016/j.ajodo.2020.03.029	Assessment of the effects of local platelet-rich fibrin injection and piezocision on orthodontic tooth movement during canine distalization	ÇAĞLI KARCI, 2021, AM J ORTHOD DENTOFACIAL ORTHOP	American Journal of Orthodontics and Dentofacial Orthopedics	23	5.75	4.75	Randomized Controlled Trial
10.1016/j.jobcr.2017.11.001	Can corticotomy (with or without bone grafting) expand the limits of safe orthodontic therapy?	BRUGNAMI F, 2018, J ORAL BIOL CRANIOFAC RES	Journal of Oral Biology and Craniofacial Research	23	3.29	1.29	Retrospective Study
10.2319/123016-940.1	Comparison of the effects of three surgical techniques on the rate of orthodontic tooth movement in a rat model	LIBRIZZI Z, 2017, ANGLE ORTHOD	The Angle orthodontist	21	2.63	1.20	Experimental study
10.2319/032414-220.1	A pilot clinical study of Class III surgical patients facilitated by improved accelerated osteogenic orthodontic treatments	WU JQ, 2015, ANGLE ORTHOD	The Angle orthodontist	21	2.10	1.22	Prospective Study
10.1111/ocr.12437	Three-dimensional assessment of accelerating orthodontic tooth movement-micro-osteoperforations vs piezocision: A randomized, parallel-group and split-mouth controlled clinical trial	ALQADASI B, 2021, ORTHODONT CRANIOFAC RES	Orthodontics & craniofacial research	20	5.00	4.13	Randomized Controlled Trial
10.1016/j.ajodo.2019.06.025	Clinical follow-up of corticotomy-accelerated Invisalign orthodontic treatment with Dental Monitoring	HANNEQUIN R, 2020, AM J ORTHOD DENTOFACIAL ORTHOP	American journal of orthodontics and dentofacial orthopedics	20	4.00	1.92	Case report
10.1016/j.ajodo.2018.11.010	A novel 3D-printed computer-assisted piezocision guide for surgically facilitated orthodontics	HOU HY, 2019, AM J ORTHOD DENTOFACIAL ORTHOP	American journal of orthodontics and dentofacial orthopedics	20	3.33	1.95	Case report
10.1186/s12903-019-0758-9	Evaluation of the levels of pain and discomfort of piezocision-assisted flapless corticotomy when treating severely crowded lower anterior teeth: a single-center, randomized controlled clinical trial	GIBREAL O, 2019, BMC ORAL HEALTH	BMC Oral Health	20	3.33	1.95	Randomized Controlled Trial

10.17219/dmp/110432	Evaluation of the effectiveness of piezocision-assisted flapless corticotomy in the retraction of four upper incisors: A randomized controlled clinical trial	AL-IMAM GMF, 2019, DENT MED PROBL	Dental and medical problems	20	3.33	1.95	Randomized Controlled Trial
10.1007/s00784-019-02887-z	Patient-reported outcomes measures (PROMs) following a piezocision-assisted versus conventional orthodontic treatments: a randomized controlled trial in adults	CHARAVET C, 2019, CLIN ORAL INVEST	Clinical oral investigations	19	3.17	1.85	Randomized Controlled Trial
10.4103/jos.JOS_112_18	Effects of mini-implant facilitated micro-osteoperforations in alleviating mandibular anterior crowding: A randomized controlled clinical trial	BANSAL M, 2019, J ORTHOD SCI	Journal of orthodontic science	19	3.17	1.85	Randomized Controlled Trial
10.4103/0970-9290.152191	Corticotomy-assisted retraction: an outcome assessment	VIJAYASHRI SAKTHI S, 2014, INDIAN J DENT RES	Indian journal of dental research	19	1.73	1.00	Prospective Study
10.1016/j.ijom.2017.02.1273	The accuracy of computer-guided piezocision: a prospective clinical pilot study	CASSETTA M, 2017, INT J ORAL MAXILLOFAC SURG	International Journal of Oral and Maxillofacial Surgery	18	2.25	1.03	Prospective Study
10.1016/j.sdentj.2018.10.003	Comparative CBCT analysis of the changes in buccal bone morphology after corticotomy and micro-osteoperforations assisted orthodontic treatment - Case series with a split mouth design	AGRAWAL AA, 2019, SAUDI DENT J	The Saudi dental journal	17	2.83	1.66	Case Series
10.1097/MD.0000000000012047	A new modified bone grafting technique for periodontally accelerated osteogenic orthodontics	MA Z, 2018, MEDICINE	Medicine	17	2.43	0.95	Prospective Study
10.1016/j.joms.2013.09.007	Accelerated Orthodontic Tooth Movement Following Le Fort I Osteotomy in a Rodent Model	YUAN H, 2014, J ORAL MAXILLOFAC SURG	Journal of Oral and Maxillofacial Surgery	17	1.55	0.89	Experimental study
10.1111/jre.12885	PTH/PTHrP in controlled release hydrogel enhances orthodontic tooth movement by regulating periodontal bone remodaling	LU W, 2021, J PERIODONTAL RES	Journal of periodontal research	16	4.00	3.30	Experimental study
